# Targeted Metabolomics for Plasma Amino Acids and Carnitines in Patients with Metabolic Syndrome Using HPLC-MS/MS

**DOI:** 10.1155/2020/8842320

**Published:** 2020-07-17

**Authors:** Li-li Gong, Song Yang, Wen Zhang, Fei-fei Han, Ling-ling Xuan, Ya-li Lv, He Liu, Li-hong Liu

**Affiliations:** Beijing Chaoyang Hospital, Capital Medical University, Beijing, China

## Abstract

Metabolic syndrome (MetS) is a health disorder characterized by metabolic abnormalities that predict an increased risk to develop cardiovascular disease (CVD) and type 2 diabetes. Biomarkers can provide an insight into the novel mechanism for MetS and can be potentially used for personalized response to therapies. We exploited a targeted HPLC-MS/MS method to characterize plasma amino acids and carnitine metabolic profile in MetS patients. A training set (40 cases and 40 controls) and validation set (80 MetS patients and 80 healthy controls) were carried out to find the metabolic profiles. We discovered two carnitine metabolites including hydroxydecanoyl carnitine and methylglutarylcarnitine. Our results indicated that the decreased level of hydroxydecanoyl carnitine and methylglutarylcarnitine may be associated with the risk of MetS. These biomarkers may improve the risk prediction and provide a novel tool for monitoring of the progression of disease and response to treatment in MetS patients.

## 1. Introduction

Metabolic syndrome (MetS) represents a cluster of metabolic abnormalities that include hypertension, central obesity, insulin resistance, and atherogenic dyslipidemia [1] and is strongly associated with an increased risk to develop cardiovascular disease (CVD) and type 2 diabetes (T2DM) [2]. The routine diagnosis of MetS is to measure the level of clinical features such as waist circumference, blood pressure, fasting glucose, triglycerides, and HDL [3]. Although these factors contribute considerably to disease risk, they may not identify individuals at risk before the disease process is well underway. Understanding the metabolic changes in patients may be an option to improve the identification of at-risk individuals and to assess their response to therapy.

The assessment of metabolite profiles in biofluids has become a powerful method for the detection of biomarker molecules and disease mechanisms. Until now, some metabolomic studies have explored the target or nontargeted metabolic changes in plasma or urine in metabolic syndrome using high-performance liquid chromatography/quadrupole time-of-flight mass spectrometry (HPLC/Q-TOF-MS) [4–7], gas chromatography mass spectrometry (GC-MS) [8], and nuclear magnetic resonance (NMR) [9]. Metabonomic technique based on HPLC-MS/MS provides an insight into the metabolic profiling and pathophysiological mechanisms [10]. Previous studies have found some biomarkers related with metabolic syndrome such as deoxysphingolipids [6], 2-hydroxybutyric acid, inositol, and D-glucose [8], and phosphatidylcholine [11]. In urine, branch-chain and aromatic amino acids (leucine, tyrosine, phenylalanine, and tryptophan) and short-chain acylcarnitine (tiglylcarnitine) [4] have showed differences between the MetS and healthy groups. Metabolic signatures of obesity and diabetes have previously been studied to gain insight into the pathophysiology of these conditions and to develop and evaluate treatments; plasma amino acids [12, 13] and carnitines [14, 15] are frequently assessed for these purposes. These results showed that amino acids' (leucine, isoleucine, valine, phenylalanine) level changed, and some carnitines such as propionylcarnitine, butyrylcarnitine, and isovalerylcarnitine increase in obesity or diabetes. Comparing the metabolites of healthy versus MetS diseased states can provide information helpful in correlating the role of amino acid and carnitine in metabolic syndrome.

Targeted metabolomics is the measurement of defined groups of chemically characterized and biochemically annotated metabolites [16]. Through the use of standards, analysis can be undertaken in a quantitative or semiquantitative manner. Therefore, the purpose of the present study was to comprehensively investigate the potential amino acids and carnitine biomarkers of metabolic syndrome. The development of novel amino acids and carnitine biomarkers for monitoring MetS would provide an additional clinical tool for diagnosis or treatment.

## 2. Materials and Methods

### 2.1. Chemicals and Reagents

HPLC-grade formic acid was obtained from Sigma-Aldrich (USA). HPLC/MS-grade formic acid, ammonium formate, and acetonitrile were acquired from Fisher Scientific (USA). The internal standards Labeled Carnitine Standards (L-carnitine (N-trimethyl-D_9_), L-acetylcarnitine (N-methyl-D_3_), L-propionylcarnitine (Nimethyl-D_3_), L-butyrylcarnitine (N-methyl-D_3_), L-isovalerylcarnitine (N-trimethyl-D_9_), L-octanoylcarnitine (N-methyl-D_3_), L-myristoylcarnitine (N-trimethyl-D_9_), L-palmitoylcarnitine (N-methyl-D_3_)) were attained from Cambridge Isotope Laboratories. Seventeen amino acid standards (alanine, valine, leucine, isoleucine, phenylalanine, methionine, proline, glycine, serine, threonine, cysteine, tyrosine, histidine, lysine, arginine, aspartic acid, glutamic acid) were purchased from Sigma-Aldrich. Eight amino acid standards (tryptophan, asparaginate, glutamine, ornithine, taurine, citrulline, cysteine, *γ*-aminobutyric acid) were gained from J&K Scientific. Ultrahigh purity water was prepared by Millipore-Q Water Purification System (Millipore, Germany).

### 2.2. Study Subjects

MetS patients and healthy controls were recruited from Beijing Chaoyang Hospital as described before [11]. Briefly, patients diagnosed with metabolic syndrome were recruited, and the healthy control (HC) groups were age and gender matched which included subjects without evidence of risk factor. A discovery plasma set with 80 subjects (40 MetSs and 40 HCs) and an independent validation set with 160 subjects (80 MetSs and 80 HCs) were used to discover and verify the differential metabolites. The Research Ethics Committees in Beijing Chaoyang Hospital approved this study (2015-12-25-2). All participants signed an informed consent document. All procedures were conducted according to the criteria set by the Declaration of Helsinki.

### 2.3. Sample Preparation

Quantitative analysis of amino acids used external standard methods. Acetonitrile (200 *μ*L) was added to 50 *μ*L plasma. After vortex mixing for 30 s, the sample was centrifuged at 16,000 × *g* at 4°C for 5 min. The supernatant (100 *μ*L) was taken and added to 600 *μ*L acetonitrile/water (1 : 1, *v*/*v*). The mixture was transferred to an autosampler vial for HPLC-MS/MS analysis.

Carnitine concentration was also measured by the HPLC-MS/MS method. An aliquot of 50 *μ*L human plasma was pipetted into a 1.5 mL Eppendorf tube and spiked with 200 *μ*L acetonitrile. After vortex mixing for 5 min, the mixture was centrifuged at 4°C for 5 min at 16,000 × *g*. Then, an aliquot of 50 *μ*L supernatant was transferred into another 1.5 mL Eppendorf tube, and 50 *μ*L internal carnitine standard (Labeled Carnitine Standards) was added for analysis.

Plasma quality control (QC) samples were employed to provide a representative “mean” sample containing all plasma samples. The QC samples were injected at the beginning and end and as every 10th injection while analyzing the study samples.

### 2.4. HPLC-MS/MS Conditions

A Shimadzu UHPLC (LC-20AD Prominence, Kyoto, Japan) coupled to a 5500 Q-Trap mass spectrometer (AB Sciex, Framingham, MA, USA) along with an electrospray ion source was used for HPLC-MS/MS analysis. An Acquity UPLC BEH Amide column (100 mm × 2.1 mm, 1.7 *μ*m, Waters) was applied for all analyses. The mobile phase was composed of A (water, which contains 0.1% formic acid) and B (acetonitrile, which contains 0.1% formic acid and 2.5 mM ammonium formate) with a gradient elution: 0-7 min, 5% A; 7-9 min, 50% A; 9-13 min, 5% A for amino acid analysis and 0-10 min, 5% A; 10-12 min, 20% A; 12-15 min, 40% A; 15-19 min, 5% A for carnitine analysis. The flow rate was set at 0.3 mL/min. The column temperature and injection volume were set at 50°C and 1 *μ*L, respectively. Mass spectrometric detection was performed using a QTRAP 5500 system. Sample ionisation was carried out in the positive mode using the Turbo Ionspray source at a source temperature of 600°C. Ion spray voltage was 5500 V, gas one had a flow of 50 units, gas two had a flow of 60 units, the curtain gas had a flow of 40 units, and the nitrogen gas setting was “medium.” The multiple reaction monitoring (MRM) mode was used, with 2 MRM transitions monitored per analyte. MRM transitions are summarized in Table S1. Data was acquired in profile mode and processed using the Analyst version 1.6.1 software.

### 2.5. Preparation of Calibration Standards

The stock solutions of internal standard were prepared in acetonitrile/water (1 : 1, *v*/*v*), respectively. Different amounts of each stock solution were mixed and diluted with acetonitrile/water (1 : 1, *v*/*v*) to prepare the series of working solutions for the analytes. Calibration curve and quality control working solutions were prepared by serially diluting stock solutions separately in acetonitrile/water (1 : 1, *v*/*v*); all of which contained appropriate concentrations of each analyte. Furthermore, 10 *μ*L working solution was spiked with 990 *μ*L blank patient plasma to obtain the required final concentrations of the calibration standard (CS).

### 2.6. Method Validation

Validation of the developed HPLC-MS/MS method was performed in the presence of plasma matrix. Linearity, limit of detection (LOD), limit of quantification (LOQ), precision, recovery, and stability were assessed.

#### 2.6.1. Linearity

Two calibration curves were generated: (1) an external standard calibration curve, made by diluting standard solutions in the mobile phase and (2) an internal standard curve, which linearity was determined for standards spiked to the QC sample at 6 different concentrations before extraction. The mean peak area of three replicate measurements at each concentration was calculated.

#### 2.6.2. Intra- and Interassay Precision

Precision was assessed for the standards at the low, medium, and high concentration levels by repeating QC sample preparation and analysis during three consecutive days. The intraday and interday precisions were calculated as %RSD of the peak area of each standard from 6 replicate assays.

#### 2.6.3. Limit of Detection (LOD) and Limit of Quantification (LOQ)

The LOD was the lowest concentration of analyte in the test sample that can be reliably distinguished from zero (signal/noise ratio ≥ 3). The LOQ was considered to be the lowest concentration of analyte that can be determined with an acceptable repeatability and trueness (signal/noise ratio ≥ 10 and RSD ≤ 20%).

#### 2.6.4. Stability

Stock solution stability of the underivatized analytes at 4°C (24 h) was investigated. For the analyte to be considered stable, the difference had to be within ±10% of the original value.

#### 2.6.5. Recovery

Recoveries were carried out for the standard at the low, medium, and high concentration levels in the QC samples spiked before and after extraction (*n* = 3). The recoveries were calculated for each standard as the ratio of the peak area in the sample spiked prior to extraction and postextraction.

### 2.7. Data Analysis

Statistical analyses were performed using IBM SPSS Statistics 21.0. Differences between these 2 groups were analyzed using either the independent *t*-test (parametric distribution) or the Mann-Whitney *U* test (nonparametric distribution) for means with continuous data. Value of *P* < 0.05 was defined as statistically significant.

PCA and OPLS-DA were constructed to determine the distributions and find the metabolic difference between the healthy control group and the metabolic syndrome group using the MetaboAnalyst 4.0. A metabolite was conservatively excluded if it had missing data in >50% of each group. For all other metabolites, missing measurements were imputed with zero filing. Metabolites with a coefficient of variation (CV higher than 30%) in QC samples after normalization were excluded. To test the discriminatory capacity of each metabolite, we performed receiver operating characteristic (ROC) analysis. We generated box plots for those metabolites with significant differences between these two groups with adjusted *P* values. Heatmaps indicate log_2_ value of fold change. Volcano plots were generated with the log_2_ fold change values and Bonferroni-adjusted *P* values.

## 3. Results

### 3.1. Assay Validation on Linearity, Accuracy and Precision, LOD and LOQ, and Stability and Recovery

Determination linear ranges, LODs, and LOQ of the standard are given in Table S3. The correlation coefficients (*R*^2^) were higher than 0.990 for all standards in their linear range, showing good linear relationship within linear ranges. All the LOD values were in range 0.003-0.02 *μ*M, and LOQ values were in range 0.001-0.07 *μ*M.

The results of intra- and interday precision analyses performed at three levels (low, medium, and high) are presented in Table S4. The averages of intra- and interday precision ranged from 1.00% to 4.22% and from 1.25% to 4.52%, respectively. These precision results were within the acceptable criteria, showing that the developed method was reliable, reproducible, and accurate for the quantitative analysis of the studied.

The stability of all analytes was over the range of 90.7-109.1%, which indicated that the stocked samples were found to be stable for 24 h at 4°C (Table S5). The results suggested that the established method was reliable and suited for large-scale sample screening.

Recoveries were evaluated at three concentration levels (low, medium, and high). As shown in Table S6, the recoveries of the analytes were within 88.1-113.7% (RSD < 15%), indicating the reliability of the developed method.

### 3.2. Screening of Differential Metabolites

**Figure 1:**
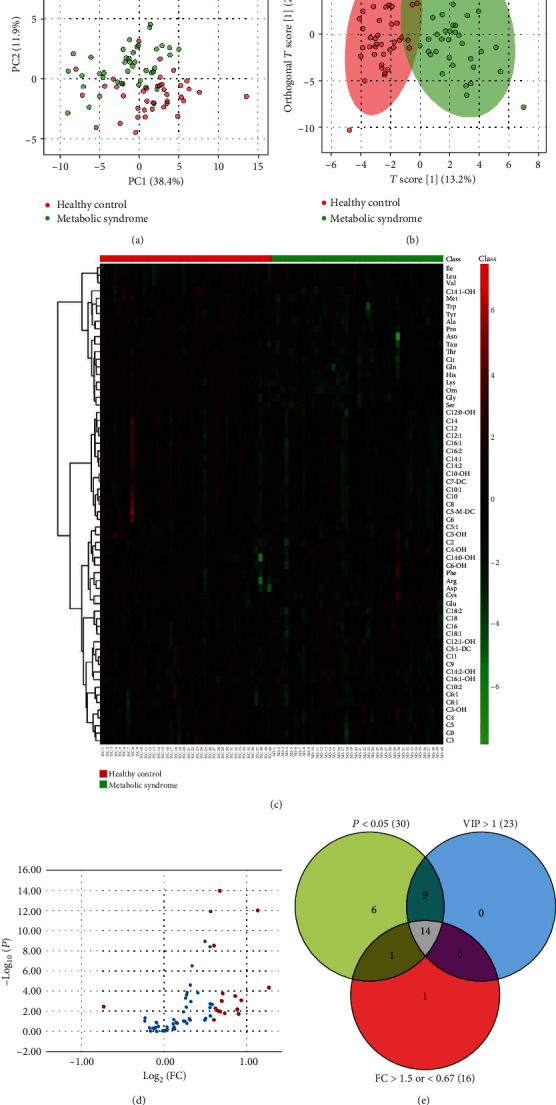
Statistical analysis for the data obtained from training set. (a) Score scatter plot for PCA of plasma metabolic profiling of the MetS and HC groups. (b) OPLS-DA of plasma samples of patients collected. (c) Heatmap showed the distribution of amino acids and carnitine metabolites between the MetS and HC groups. Volcano plot of different metabolites. (d) The volcano plot is a combination of fold change and *t*-tests. The *x*-axis is log_2_ (FC), *y*-axis is -log_10_ (*P* value). The red dots are fold change > 1.5 or <0.67. (e) Venn diagram of VIP, adjusted *P*, and fold change results.

Multivariate analysis by PCA and OPLS-DA analysis showed a clear intergroup separation in metabolites between the MetS patients and HC groups (Figures 1(a) and 1(b)). Amino acid and carnitine metabolites were displayed as a heatmap (Figure 1(c)). The volcano plot was based on *P* value from a *t*-test and the fold change values (Figure 1(d)). This plot is colored such that those points having a fold change > 1.5 or <0.67 are shown in red. Together, a total of 14 metabolites with VIP threshold (VIP > 1), *P* value (*P* < 0.05) with a false discovery rate (FDR) < 0.05, and fold change > 1.5 or <0.67 were selected as metabolite markers and summarized in Figure 1(e) and Table S1.

### 3.3. Validation and Evaluation of Potential Biomarkers

**Figure 2:**
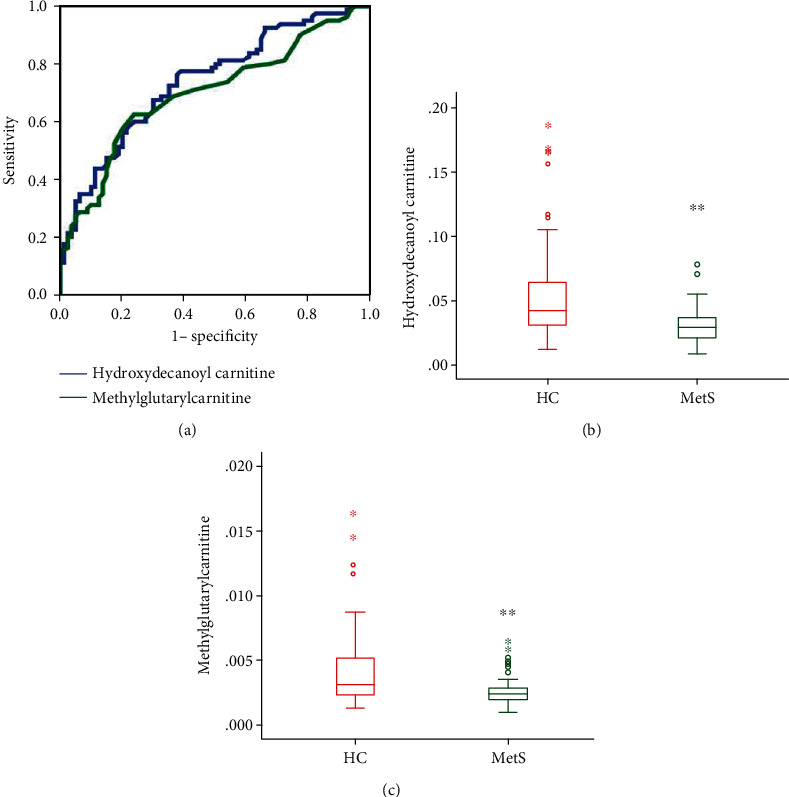
(a) Receiver operating characteristic (ROC) curve of 2 carnitines from patients with metabolic syndrome in validation phase. (b, c) Box plots showing significant differential metabolite changes in plasma between the healthy control and MetS groups. Boxes show interquartile ranges, and lines show medians. ^∗^*P* < 0.05; ^∗∗^*P* < 0.01, compared with the control group.

We quantitatively examined the levels of 14 metabolites in plasma samples, including 4 amino acids and 10 carnitine metabolites. The candidate metabolites were validated through external/interior standard method. Receiver operating characteristic (ROC) curve was exploited based on the results of the area under the curve (AUC). There are significant differences about the concentration of asparagine, glutamine, and ornament. However, the ROC curves showed that 0.26, 0.593, and 0.367 which suggested they cannot be used to diagnose patients with and without MetS. Two of carnitines revealed satisfactory diagnostic values with area under ROC curve (AUC) more than 0.7. The ROC curves of the individual biomarkers from MetS patient invalidation phase are shown in Figure 2(a). Box plots are provided in Figures 2(b) and 2(c), demonstrating fluctuations in single metabolites in MetS patients. Hydroxydecanoyl carnitine and methylglutarylcarnitine were significantly decreased in MetS patients when compared with the HC group.

### 3.4. Impact of Other Factors on Potential Biomarkers

**Figure 3:**
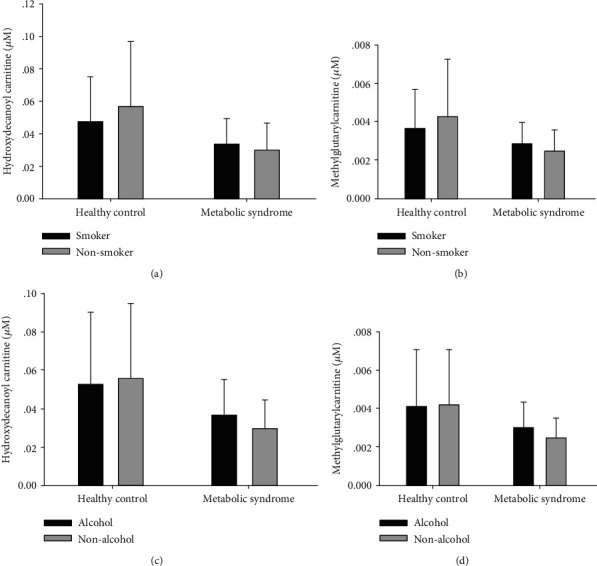
The influence of smoking and alcohol consumption on the potential biomarkers. Comparison of 2 potential biomarkers between “smoker” and “non-smoker” groups and “alcohol” and “non-alcohol” groups with the healthy and MetS participants.

We have collected other factors, which might influence the levels of these 2 potential biomarkers, including smoking and alcohol consumption. As we have shown before, the smoking statuses and alcohol consumption were significantly different between the metabolic syndrome groups and healthy controls (*P* < 0.05) [11]. No statistical differences were observed in the potential biomarkers hydroxydecanoyl carnitine and methylglutarylcarnitine and among different smoking statuses and alcohol consumption, suggesting that plasma concentration of carnitine is not affected by smoking and/or alcohol consumption (Figure 3).

## 4. Discussion

Previous studies have found some amino acid and carnitine biomarkers related with metabolic syndrome in urine such as nicotinuric acid [7], some amino acids (leucine, tyrosine, phenylalanine, and tryptophan), tiglylcarnitine [4], and also BCAAs [17], aromatic amino acids, lysine, and its metabolite, alpha-aminoadipate [18] in plasma. Current MetS metabolomic studies have made promising progress, but still have some shortcomings, such as a lack of validation.

**Figure 4:**
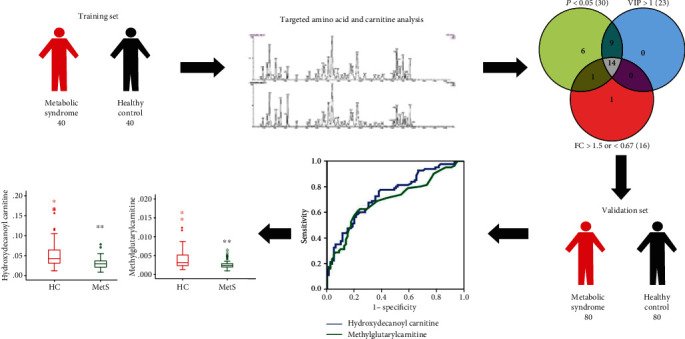
The study flow chart. A targeted metabolomic analysis in human plasma samples to identify differences of amino acid and carnitine in metabolic features between HC and MetS patients in the training set (40 MetSs and 40 HCs). A total of 14 metabolites with VIP threshold (VIP > 1), *P* value (*P* < 0.05) with a false discovery rate (FDR) < 0.05, and fold change > 1.5 or <0.67 were selected as metabolite markers. Then, there is another validation analysis to further evaluate changes in the levels of potential biomarkers between MetS patients and HC (80 MetSs and 80 HCs). Two of carnitines revealed satisfactory diagnostic values with area under ROC curve more than 0.7. The levels of hydroxydecanoyl carnitine and methylglutarylcarnitine were significantly decreased in MetS patients when compared with the HC group.

In the present study, we performed a targeted metabolomic analysis in human plasma samples to identify differences of amino acid and carnitine in metabolic features between HC and MetS patients by HPLC-MS/MS. We also employed another validation analysis to further evaluate changes in the levels of potential biomarkers between MetS patients and HC. The present study showed a clear decrease in plasma carnitine (hydroxydecanoyl carnitine and methylglutarylcarnitine) in MetS patients (Figure 4).

Carnitine is a key metabolite related to obesity, due to its involvement in fatty acid metabolism [19]. Fatty acids must bind with a carnitine molecule to be metabolized through *β*-oxidation in the mitochondrion or the peroxisome. Thus, higher plasma FFAs in obesity may require more carnitine for efficient *β*-oxidation. Consequently, the amount of carnitine in cells is an important factor regulating the process of *β*-oxidation [20]. Our MetS patients exhibited significant reduction of plasma hydroxydecanoyl carnitine and methylglutarylcarnitine levels. These findings are in accord with the results of the earlier studies which have shown reduced plasma carnitine in obese humans [21]. It has been reported that supplementation of carnitine can decrease body weight gain, adiposity, insulin serum concentration, and TAG liver content and improve insulin resistance in obese Zucker rats [22]. This research is potentially explaining why serum levels of carnitine were decreased in MetS patients in this study.

Previous studies of amino acid or carnitine on metabolic syndrome have mainly focused on screening differential metabolites. In this study, the 4 amino acids and 10 carnitines were discovered from the training set and were further evaluated in a validation set. We found two carnitines that showed good potential to distinguish MetS patients from healthy individuals: hydroxydecanoyl carnitine and methylglutarylcarnitine. ROC curves were calculated for each of the metabolites, with AUC values 0.74 and 0.70 in validation set (Figure 2). Hydroxydecanoyl carnitine and methylglutarylcarnitine could potentially serve as a marker to predict the risk of MetS and offer an option on the treatment of metabolic syndrome.

## 5. Conclusions

In summary, we employed a targeted metabolomic analysis to identify plasma metabolic profile in MetS patients. We found two carnitines (hydroxydecanoyl carnitine and methylglutarylcarnitine) based on the screening and validation procedure. Carnitine is essential for the transfer of long-chain fatty acids across the inner mitochondrial membrane for subsequent *β*-oxidation that plays an essential role in energy metabolism [23]. The carnitine biomarker we found in this study provides a possibility for future diagnostic or therapeutic development.

## Data Availability

The [DATA TYPE] data used to support the findings of this study are available from the corresponding author upon request.

## Supplementary Materials

Supplementary Materials Table S1: fourteen identified differential metabolites between MetS and healthy subjects. Table S2: MRM transitions for amino acids and carnitine. Table S3: validation parameters (linear range, limit of detection (LOD), and limit of quantification (LOQ)) for the analyzed compounds. Table S4: intraday and interday assay precision results of amino acid and carnitine. Table S5: summary of stability of stock solutions (24 h at 4°C). Table S6: recovery results of amino acid and carnitine.
