# Supplementary material for: Targeted Metabolomics for Plasma Amino Acids and Carnitines in Patients with Metabolic Syndrome Using HPLC-MS/MS
Source: Dis Markers. 2020 Jul 17;2020:8842320. doi: 10.1155/2020/8842320 (PMC7383313; doi:10.1155/2020/8842320)
Supplement: Supplementary Materials — Table S1: fourteen identified differential metabolites between MetS and healthy subjects. Table S2: MRM transitions for amino acids and carnitine. Table S3: validation parameters (linear range, limit of detection (LOD), and limit of quantification (LOQ)) for the analyzed compounds. Table S4: intraday and interday assay precision results of amino acid and carnitine. Table S5: summary of stability of stock solutions (24 h at 4°C). Table S6: recovery results of amino acid and carnitine. [file 8842320.f1.docx]

**Supporting Information**

**Targeted metabolomics for plasma amino acids and carnitines in patients with Metabolic Syndrome using HPLC-MS/MS**

Li-li Gong*, Song Yang, Wen Zhang, Ling-ling Xuan, Fei-fei Han, Ya-li Lv, He Liu, Li-hong Liu*

*Beijing Chao-Yang Hospital, Capital Medical University, Beijing, China*

*Corresponding authors: Beijing Chaoyang Hospital, Capital Medical University, 8 Gongren Tiyuchang Nanlu, Beijing 100020, China, Tel: 86-10-85231464;

*E-mail address*: gonglili@126.com, liulihong@bjcyh.com

**Table of contents**

Table S1

Table S2

Table S3

Table S4

Table S5

Table S6

Table S1. Fourteen Identified Differential Metabolites between MetS and Healthy Subjects

| Index | Metabolites | m/z | VIP | Fold change  MetS vs HC | *P* value |
| --- | --- | --- | --- | --- | --- |
| 1 | Asparagine (Asp) | 132.1179 | 2.0563 | 2.4062 | 1.77E-12 |
| 2 | Citrulline (Cit) | 175.1857 | 2.0323 | 2.1932 | 1.19E-12 |
| 3 | Glutarnine (Gln) | 146.1445 | 1.5230 | 1.5985 | 1.42E-14 |
| 4 | Ornament (Orn) | 132.1610 | 1.3297 | 1.5212 | 3.34E-09 |
| 5 | Hydroxytetradecadienylcarnitine(C14:2-OH) | 384.2744 | 2.2790 | 1.5419 | 0.008558 |
| 6 | Tetradecadienylcarnitine(C14:2) | 368.2795 | 1.7465 | 1.8226 | 0.001193 |
| 7 | Octanoylcarnitine(C8) | 288.2169 | 1.6243 | 1.6054 | 0.002369 |
| 8 | [Hydroxydecanoyl carnitine](http://www.hmdb.ca/metabolites/HMDB61636) (C10-OH) | 331.4476 | 1.5606 | 1.6054 | 0.003055 |
| 9 | Hydroxytetradecenoylcarnitine(C14:1-OH) | 386.2901 | 1.5146 | 1.8730 | 0.011039 |
| 10 | Hexadecadienylcarnitine(C16:2) | 396.3108 | 1.4951 | 1.6242 | 0.003163 |
| 11 | Methylglutarylcarnitine(C5-M-DC) | 289.3248 | 1.4811 | 1.6375 | 0.000699 |
| 12 | Decanoylcarnitine(C10) | 316.2482 | 1.4019 | 1.8564 | 0.014473 |
| 13 | Hexanoylcarnitine(C6) | 260.1856 | 1.2258 | 0.6067 | 0.013527 |
| 14 | Dodecanoylcarnitine (C12) | 344.2795 | 1.0374 | 1.6654 | 0.015565 |

Table S2. MRM Transitions for amino acids and carnitine.

| Index | Name | Parent Ion | Product Ion | DP (V) | CE (V) | |
| --- | --- | --- | --- | --- | --- | --- |
| 1 | Alanine | 89.9 | 44.0 | 25 | 14 |  |
| 2 | Arginine | 175.0 | 70.1 | 40 | 30 |  |
| 3 | Asparagine | 133.1 | 87.1 | 40 | 14 |  |
| 4 | Aspartic acid | 134.0 | 88.0 | 40 | 18 |  |
| 5 | Cystine | 241.0 | 152.1 | 40 | 17 |  |
| 6 | Glutamine | 147.1 | 84.0 | 40 | 22 |  |
| 7 | Glutamic acid | 148.0 | 102.0 | 40 | 15 |  |
| 8 | Glycine | 76.1 | 30.0 | 40 | 15 |  |
| 9 | Histidine | 156.0 | 110.1 | 40 | 19 |  |
| 10 | Isoleucine | 132.0 | 86.1 | 40 | 14 |  |
| 11 | Leucine | 132.1 | 86.1 | 40 | 14 |  |
| 12 | Lysine | 147.0 | 84.1 | 40 | 22 |  |
| 13 | Methionine | 149.9 | 104.0 | 40 | 15 |  |
| 14 | Phenylalanine | 166.0 | 120.1 | 40 | 17 |  |
| 15 | Proline | 116.0 | 70.2 | 40 | 18 |  |
| 16 | Serine | 106.0 | 60.1 | 40 | 15 |  |
| 17 | Threonine | 120.0 | 74.0 | 40 | 15 |  |
| 18 | Tryptophan | 205.1 | 188.0 | 40 | 23 |  |
| 19 | Tyrosine | 182.0 | 136.0 | 40 | 18 |  |
| 20 | Valine | 117.9 | 72.1 | 40 | 20 |  |
| 21 | Ornithine | 133.0 | 70.0 | 40 | 30 |  |
| 22 | Taurine | 126.2 | 44.1 | 40 | 31 |  |
| 23 | Citrulline | 176.0 | 113.2 | 40 | 20 |  |
| 24 | Carnitine (C0) | 162.2 | 85.0 | 100 | 20 |  |
| 25 | Acetylcarnitine (C2) | 204.2 | 85.0 | 100 | 20 |  |
| 26 | Propionylcarnitine (C3) | 218.2 | 85.0 | 100 | 20 |  |
| 27 | Butenoylcarnitine (C4:1) | 230.2 | 85.0 | 100 | 20 |  |
| 28 | Butyrylcarnitine (C4) | 232.2 | 85.0 | 100 | 20 |  |
| 29 | Hydroxypropionylcarnitine (C3-OH) | 234.2 | 85.0 | 100 | 20 |  |
| 30 | Valerylcarnitine (C5) | 246.2 | 85.0 | 100 | 20 |  |
| 31 | Hydroxybutyrylcarnitine (C4-OH) | 248.2 | 85.0 | 100 | 20 |  |
| 32 | Hexenoylcarnitine (C6:1) | 258.2 | 85.0 | 100 | 20 |  |
| 33 | Hexanoylcarnitine (C6) | 260.2 | 85.0 | 100 | 20 |  |
| 34 | Octanoylcarnitine (C8) | 288.2 | 85.0 | 100 | 20 |  |
| 35 | Decadienylcarnitine (C10:2) | 312.2 | 85.0 | 100 | 20 |  |
| 36 | Decenoylcarnitine (C10:1) | 314.2 | 85.0 | 100 | 20 |  |
| 37 | Decanoylcarnitine(C10) | 316.2 | 85.0 | 100 | 20 |  |
| 38 | Dodecenoylcarnitine (C12:1) | 342.2 | 85.0 | 100 | 20 |  |
| 39 | Dodecanoylcarnitine (C12) | 344.2 | 85.0 | 100 | 20 |  |
| 40 | Tetradecadienylcarnitine (C14:2) | 368.2 | 85.0 | 100 | 20 |  |
| 41 | Tetradecenoylcarnitine (C14:1) | 370.2 | 85.0 | 100 | 20 |  |
| 42 | Tetradecanoylcarnitine (C14) | 372.3 | 85.0 | 100 | 20 |  |
| 43 | Hydroxytetradecadienylcarnitine (C14:2-OH) | 384.2 | 85.0 | 100 | 20 |  |
| 44 | Hydroxytetradecenoylcarnitine (C14:1-OH) | 386.2 | 85.0 | 100 | 20 |  |
| 45 | Hexadecadienylcarnitine (C16:2) | 396.2 | 85.0 | 100 | 20 |  |
| 46 | Hexadecenoylcarnitine (C16:1) | 398.2 | 85.0 | 100 | 20 |  |
| 47 | Hexadecanoylcarnitine (C16) | 400.2 | 85.0 | 100 | 20 |  |
| 48 | Hydroxyhexadecenoylcarnitine (C16:1-OH) | 414.3 | 85.0 | 100 | 20 |  |
| 49 | Hydroxyhexadecanoylcarnitine(C16-OH) | 416.2 | 85.0 | 100 | 20 |  |
| 50 | Octadecenoylcarnitine (C18:1) | 426.2 | 85.0 | 100 | 20 |  |
| 51 | Octadecanoylcarnitine (C18) | 428.2 | 85.0 | 100 | 20 |  |
| 52 | Octadecadienylcarnitine (C18:2) | 440.2 | 85.0 | 100 | 20 |  |
| 53 | Hydroxyoctadecenoylcarnitine (C18-OH) | 442.2 | 85.0 | 100 | 20 |  |
| 54 | Carnitine-D9 | 171.2 | 85.0 | 100 | 20 |  |
| 55 | Acetylcarnitine-D3 | 207.2 | 85.0 | 100 | 20 |  |
| 56 | Propionylcarnitine-D3 | 221.2 | 85.0 | 100 | 20 |  |
| 57 | Butyrylcarnitine-D3 | 235.2 | 85.0 | 100 | 20 |  |
| 58 | Valerylcarnitine-D9 | 255.2 | 85.0 | 100 | 20 |  |
| 59 | Octanoylcarnitine-D3 | 291.2 | 85.0 | 100 | 20 |  |
| 60 | Palmitoylcarnitine-D3 | 403.2 | 85.0 | 100 | 20 |  |
| 61 | Myristoylcarnitine-D9 | 381.3 | 85.0 | 100 | 20 |  |

Table S3. Validation parameters (linear range, limit of detection (LOD) and limit of quantification (LOQ) for the analyzed compounds.

| Index | Name | Linear range  μM | R^2^ | LOD | LOQ |
| --- | --- | --- | --- | --- | --- |
| 1 | Alanine | 4-500 | 0.995 | 0.010 | 0.030 |
| 2 | Arginine | 4-200 | 0.996 | 0.003 | 0.010 |
| 3 | Asparagine | 4-200 | 0.992 | 0.010 | 0.020 |
| 4 | Aspartic acid | 4-200 | 0.991 | 0.020 | 0.050 |
| 5 | Cystine | 4-200 | 0.993 | 0.010 | 0.040 |
| 6 | Glutamine | 4-1000 | 0.994 | 0.010 | 0.020 |
| 7 | Glutamic acid | 4-200 | 0.995 | 0.010 | 0.030 |
| 8 | Glycine | 4-200 | 0.993 | 0.020 | 0.070 |
| 9 | Histidine | 4-200 | 0.995 | 0.010 | 0.020 |
| 10 | Isoleucine | 4-200 | 0.998 | 0.003 | 0.010 |
| 11 | Leucine | 4-200 | 0.997 | 0.003 | 0.010 |
| 12 | Lysine | 4-200 | 0.996 | 0.010 | 0.020 |
| 13 | Methionine | 4-200 | 0.998 | 0.010 | 0.020 |
| 14 | Phenylalanine | 4-200 | 0.999 | 0.003 | 0.010 |
| 15 | Proline | 4-200 | 0.999 | 0.003 | 0.010 |
| 16 | Serine | 4-200 | 0.992 | 0.020 | 0.050 |
| 17 | Threonine | 4-200 | 0.998 | 0.002 | 0.005 |
| 18 | Tryptophan | 4-200 | 0.997 | 0.002 | 0.005 |
| 19 | Tyrosine | 4-200 | 0.996 | 0.002 | 0.005 |
| 20 | Valine | 4-200 | 0.995 | 0.003 | 0.010 |
| 21 | Ornithine | 4-200 | 0.994 | 0.003 | 0.010 |
| 22 | Taurine | 4-200 | 0.997 | 0.010 | 0.020 |
| 23 | Citrulline | 4-200 | 0.996 | 0.003 | 0.010 |
| 24 | Carnitine-D9 | 1.25-50 | 0.994 | 0.0015 | 0.0050 |
| 25 | Acetylcarnitine-D3 | 1.25-50 | 0.996 | 0.0010 | 0.0030 |
| 26 | Propionylcarnitine-D3 | 1.25-50 | 0.995 | 0.0007 | 0.0020 |
| 27 | Butyrylcarnitine-D3 | 1.25-50 | 0.999 | 0.0007 | 0.0020 |
| 28 | Valerylcarnitine-D9 | 1.25-50 | 0.995 | 0.0015 | 0.0050 |
| 29 | Octanoylcarnitine-D3 | 1.25-50 | 0.997 | 0.0010 | 0.0030 |
| 30 | Palmitoylcarnitine-D3 | 1.25-50 | 0.998 | 0.0006 | 0.0020 |
| 31 | Myristoylcarnitine-D9 | 1.25-50 | 0.995 | 0.0004 | 0.0010 |

Table S4. Intra-day and Inter-day assay precision results of amino acid and carnitine.

| Index | Name | Intra-day precision RSD% | | | Inter-day precision RSD% | | |
| --- | --- | --- | --- | --- | --- | --- | --- |
|  |  | Low level | Midum level | High level | Low level | Midum level | High level |
| 1 | Alanine | 1.28% | 2.47% | 1.55% | 2.21% | 1.88% | 2.24% |
| 2 | Arginine | 2.15% | 2.52% | 1.29% | 2.85% | 2.15% | 2.97% |
| 3 | Asparagine | 3.02% | 2.95% | 1.37% | 2.19% | 3.02% | 3.12% |
| 4 | Aspartic acid | 1.52% | 1.65% | 1.00% | 1.95% | 2.35% | 1.85% |
| 5 | Cystine | 1.62% | 1.82% | 2.31% | 1.86% | 1.99% | 2.08% |
| 6 | Glutamine | 1.24% | 1.87% | 2.58% | 3.06% | 1.67% | 2.03% |
| 7 | Glutamic acid | 3.52% | 2.05% | 2.64% | 1.25% | 3.01% | 2.88% |
| 8 | Glycine | 4.05% | 1.99% | 2.91% | 1.95% | 2.35% | 3.01% |
| 9 | Histidine | 3.01% | 1.09% | 2.21% | 1.86% | 1.99% | 2.82% |
| 10 | Isoleucine | 1.95% | 3.06% | 2.34% | 2.24% | 2.75% | 2.15% |
| 11 | Leucine | 1.64% | 3.02% | 2.61% | 2.95% | 1.98% | 2.42% |
| 12 | Lysine | 2.85% | 2.81% | 2.52% | 2.87% | 1.62% | 2.05% |
| 13 | Methionine | 2.15% | 2.58% | 2.97% | 2.01% | 1.82% | 2.55% |
| 14 | Phenylalanine | 3.01% | 1.89% | 3.05% | 3.28% | 1.87% | 2.33% |
| 15 | Proline | 1.95% | 1.92% | 3.12% | 3.15% | 3.21% | 2.52% |
| 16 | Serine | 1.89% | 1.25% | 1.69% | 4.02% | 3.52% | 2.91% |
| 17 | Threonine | 1.21% | 1.98% | 1.71% | 4.09% | 3.07% | 1.75% |
| 18 | Tryptophan | 1.65% | 3.56% | 1.29% | 2.97% | 2.58% | 2.15% |
| 19 | Tyrosine | 1.83% | 1.69% | 2.05% | 3.05% | 2.29% | 3.24% |
| 20 | Valine | 2.85% | 1.84% | 1.28% | 2.69% | 2.15% | 2.87% |
| 21 | Ornithine | 2.67% | 2.01% | 1.25% | 3.12% | 2.95% | 3.25% |
| 22 | Taurine | 3.62% | 1.28% | 1.92% | 3.17% | 1.75% | 3.61% |
| 23 | Citrulline | 3.69% | 4.12% | 2.83% | 4.52% | 2.18% | 3.91% |
| 24 | Carnitine-D9 | 1.25% | 1.53% | 2.11% | 2.31% | 1.90% | 2.06% |
| 25 | Acetylcarnitine-D3 | 1.52% | 2.23% | 1.96% | 3.16% | 2.12% | 2.45% |
| 26 | Propionylcarnitine-D3 | 2.18% | 2.29% | 3.12% | 4.07% | 2.33% | 2.95% |
| 27 | Butyrylcarnitine-D3 | 3.42% | 2.15% | 2.65% | 3.18% | 2.52% | 4.01% |
| 28 | Valerylcarnitine-D9 | 2.18% | 2.95% | 3.12% | 2.78% | 2.72% | 3.09% |
| 29 | Octanoylcarnitine-D3 | 3.21% | 1.75% | 3.85% | 3.06% | 1.96% | 2.51% |
| 30 | Palmitoylcarnitine-D3 | 4.22% | 2.16% | 3.02% | 4.05% | 2.54% | 3.85% |
| 31 | Myristoylcarnitine-D9 | 2.98% | 3.24% | 3.25% | 3.65% | 2.94% | 3.27% |

Table S5. Summary of stability of stock solutions (24 hr at 4°C).

| Index | Name | Stability% | | |
| --- | --- | --- | --- | --- |
|  |  | Low level | Midum level | High level |
| 1 | Alanine | 109.10% | 105.50% | 91.20% |
| 2 | Arginine | 108.80% | 104.60% | 92.80% |
| 3 | Asparagine | 102.40% | 99.52% | 94.60% |
| 4 | Aspartic acid | 99.70% | 95.80% | 92.70% |
| 5 | Cystine | 91.50% | 95.20% | 96.70% |
| 6 | Glutamine | 98.80% | 92.80% | 95.60% |
| 7 | Glutamic acid | 95.20% | 98.80% | 101.20% |
| 8 | Glycine | 90.70% | 97.20% | 102.50% |
| 9 | Histidine | 105.20% | 101.80% | 104.20% |
| 10 | Isoleucine | 98.40% | 95.50% | 92.70% |
| 11 | Leucine | 95.70% | 92.30% | 91.80% |
| 12 | Lysine | 105.20% | 94.80% | 92.50% |
| 13 | Methionine | 102.10% | 103.50% | 94.40% |
| 14 | Phenylalanine | 105.40% | 95.60% | 94.50% |
| 15 | Proline | 104.20% | 95.80% | 98.90% |
| 16 | Serine | 109.80% | 95.50% | 102.90% |
| 17 | Threonine | 98.60% | 95.80% | 96.80% |
| 18 | Tryptophan | 97.20% | 99.40% | 105.20% |
| 19 | Tyrosine | 101.50% | 95.70% | 94.80% |
| 20 | Valine | 104.50% | 102.20% | 96.80% |
| 21 | Ornithine | 105.40% | 102.10% | 98.60% |
| 22 | Taurine | 92.90% | 98.50% | 102.40% |
| 23 | Citrulline | 107.60% | 99.80% | 91.90% |
| 24 | Carnitine-D9 | 105.40% | 101.20% | 99.90% |
| 25 | Acetylcarnitine-D3 | 96.40% | 97.20% | 105.20% |
| 26 | Propionylcarnitine-D3 | 91.80% | 95.50% | 106.10% |
| 27 | Butyrylcarnitine-D3 | 92.50% | 103.20% | 108.90% |
| 28 | Valerylcarnitine-D9 | 105.80% | 100.90% | 98.10% |
| 29 | Octanoylcarnitine-D3 | 103.96% | 96.90% | 92.80% |
| 30 | Palmitoylcarnitine-D3 | 105.50% | 94.80% | 93.20% |
| 31 | Myristoylcarnitine-D9 | 107.50% | 96.70% | 92.50% |

Table S6. Recovery results of amino acid and carnitine.

| Index | Name | Recovery% | | |
| --- | --- | --- | --- | --- |
|  |  | Low level | Midum level | High level |
| 1 | Alanine | 102.5% | 99.6% | 96.7% |
| 2 | Arginine | 91.3% | 101.3% | 98.6% |
| 3 | Asparagine | 95.3% | 99.5% | 103.2% |
| 4 | Aspartic acid | 97.2% | 96.5% | 94.6% |
| 5 | Cystine | 89.5% | 95.5% | 97.4% |
| 6 | Glutamine | 113.7% | 108.5% | 98.7% |
| 7 | Glutamic acid | 94.6% | 95.3% | 96.1% |
| 8 | Glycine | 94.2% | 97.2% | 94.3% |
| 9 | Histidine | 88.1% | 94.6% | 92.5% |
| 10 | Isoleucine | 104.5% | 98.4% | 94.9% |
| 11 | Leucine | 89.0% | 95.7% | 96.6% |
| 12 | Lysine | 93.2% | 103.5% | 100.2% |
| 13 | Methionine | 101.1% | 98.4% | 92.6% |
| 14 | Phenylalanine | 98.6% | 96.8% | 101.6% |
| 15 | Proline | 92.5% | 95.8% | 95.7% |
| 16 | Serine | 96.1% | 106.1% | 96.3% |
| 17 | Threonine | 94.5% | 98.2% | 97.2% |
| 18 | Tryptophan | 95.3% | 96.8% | 94.2% |
| 19 | Tyrosine | 95.8% | 104.3% | 101.5% |
| 20 | Valine | 97.4% | 94.8% | 98.5% |
| 21 | Ornithine | 96.2% | 96.5% | 98.2% |
| 22 | Taurine | 93.8% | 96.6% | 94.8% |
| 23 | Citrulline | 97.2% | 102.6% | 98.4% |
| 24 | Carnitine-D9 | 93.2% | 98.4% | 96.2% |
| 25 | Acetylcarnitine-D3 | 94.5% | 99.5% | 95.8% |
| 26 | Propionylcarnitine-D3 | 98.5% | 108.1% | 98.8% |
| 27 | Butyrylcarnitine-D3 | 103.1% | 101.6% | 98.7% |
| 28 | Valerylcarnitine-D9 | 94.9% | 99.6% | 99.5% |
| 29 | Octanoylcarnitine-D3 | 93.7% | 98.4% | 95.5% |
| 30 | Palmitoylcarnitine-D3 | 95.6% | 98.7% | 96.5% |
| 31 | Myristoylcarnitine-D9 | 93.2% | 101.5% | 95.6% |
